# Knowledge About Epidural Analgesia Among Women in Al-Qassim Province of Saudi Arabia

**DOI:** 10.7759/cureus.38420

**Published:** 2023-05-02

**Authors:** Mohammed Geddawy, Salem S Alkraydees, Mohammed M Almadhi, Yazeed Alhabeeb, Raghad Albarrak, Juri Alghofaili, Abdulrahman Aljurbua

**Affiliations:** 1 Anesthesiology, King Saud Medical City, Riyadh, SAU; 2 General Surgery, King Saud Medical City, Riyadh, SAU; 3 Anesthesiology, King Fahad Specialist Hospital, Buraydah, SAU; 4 Medicine, Qassim University, Buraydah, SAU

**Keywords:** awareness, saudi adults, anesthesia knowledge, epidural analgesia, anesthesia

## Abstract

Introduction: Epidural analgesia is one of the most effective and fast anesthesia techniques to relieve labor pain. The technique involves inserting a needle in the epidural space that covers the spinal cord, providing a full block of sensation in the lower part of the abdomen and the lower limbs that starts seconds to minutes after the administration, while the effects last for a couple of hours after. This study assessed women's knowledge of epidural analgesia in the Al-Qassim Province of Saudi Arabia.

Methods: The study was conducted through a descriptive cross-sectional survey distributed among citizens of Qassim Province, Kingdom of Saudi Arabia, from October 2022 to February 2023. The respondents were selected randomly via social media advertising, and only women older than 18 were allowed to participate in the survey.

Results: Out of 520 responses, 483 met the study criteria. This study found significant associations between awareness levels of epidural analgesia and pregnancy history. Women who had previous histories reported epidural analgesia as the most effective means of pain control (p < 0.001), postpartum back pain as the major complication (p = 0.025), being used by the majority of other women (p = 0.022), and the potential for epidural analgesia to yield complications (p < 0.001). This means that other studies are required to explore more such associations to widen the scope of this field of study.

Conclusion: This study highlights the mixed awareness levels of epidural analgesia among Saudi women from the Qassim area. The awareness provided by clinics or hospitals is limited, and further research is required to explore the awareness levels of epidural analgesia. Overall, the study highlights the need for a concerted effort by healthcare providers to improve the awareness and uptake of epidural analgesia in the Qassim area and beyond.

## Introduction

Giving birth is a painful, challenging experience that mothers go through; it also involves other feelings like fear and happiness. So, there are many interventions that we can do to facilitate this experience. The painful sensation during labor results from multiple psychosocial and physiologic factors which induce this pain, which the nerve cell interprets as a nociceptive labor stimulus. Epidural analgesia can be very helpful in reducing the intensity of that pain [1].

Epidural analgesia is one of the most effective anesthesia techniques to relieve labor pain [2]. Epidural analgesia is a fast way to manage pain during childbirth. The technique involves inserting a needle in the epidural space that covers the spinal cord. This method gives a block of sensation in the lower part of the abdomen and the lower limbs [3]. The effect of analgesia starts seconds to minutes after administration [4]. Even though it is helpful and easy, many women refuse to receive it during labor, and that is because of their lack of awareness [5]. On the other hand, a 2014 study was conducted in Nigeria about epidural analgesia among pregnant women. A survey about epidural analgesia was distributed among 420 pregnant ladies, and the results show the following: 405 responses out of 420 responses (94.6%) were complete responses to all the questions asked. Three hundred twenty-two responses, which equals 79.5%, were unaware of epidural analgesia. Of the 83 people (20.9%) knowledgeable about epidural analgesia, 22.9% knew it is used for relieving pain during labor, and 20 (24.1%) learned some information about epidural analgesia from other health workers. Of the many responses, 310 (76.5%) have no problem with epidural analgesia. The previous labor experience and the level of education are statistically significantly related to the awareness and acceptance of this type of analgesia (p = 0.000). In conclusion, there is poor awareness and acceptance of epidural analgesia among pregnant ladies in the region [6].

Another study was conducted in King Abdulaziz Medical City in Jeddah to determine pregnant women's awareness of Epidural analgesia (EPA). The study involved 105 women. They found that 25 (23.8%) respondents revealed a complete lack of knowledge regarding EPA, 63 (60%) showed minimal awareness, and 17 (16.2%) were aware of EPA from various sources [7].

Many studies have found that women have a low level of knowledge about epidural analgesia. Most medical centers do not provide appropriate prenatal education about EPA. Also, in some medical centers, women with little information are required to make a fast and important decision, and this stressful experience may result in a negative birth experience. It is essential to educate women about different pain relief modalities. Therefore, this study aimed to assess women's knowledge of epidural analgesia in the Al-Qassim Province of Saudi Arabia.

## Materials and methods

Study design

The study was a descriptive, cross-sectional, questionnaire-based survey.

Study setting

The study was conducted among citizens of Al-Qassim Province, Kingdom of Saudi Arabia, from October 2022 to February 2023. The respondents were selected randomly via social media advertising.

Sample size

The sample size was determined in light of previous literature and theoretical support. According to these criteria, the population size (N), following the finite population correction (FPC) factor, was generally expected to be almost 1,000,000. This criterion is used as the sample without replacement for more than 5% of a finite population. Thus, the hypothesized frequency (%) of the outcome factor in the population (p) was determined following the FPC criteria. The expected range was 50 outcome factors in the population (p) with 5% of the hypothesized frequency [8]. However, the confidence limit (d) was determined to be up to 5%, with the design effect (Deff) for cluster surveys expected to be 1. After deducting the confidence limits (d), the remaining confidence level was 95% to determine the normality of the sample [9]. Thus, the finalized sample size for the current study was 384, following the abovementioned criteria.

Data collection methods

The study was conducted using an online, self-administered questionnaire created via Google Forms. The generated link was randomly shared on social media platforms by the researchers (i.e., Facebook, WhatsApp, Telegram, and Twitter). The aim of the study was clearly explained in the interface.

A validated questionnaire was used based on previous studies [10,11]. The questionnaire contained demographics, age, pregnancy history; different aspects of epidural analgesia; and the role of medical staff and organizations in disseminating information on epidural analgesia among the Al Qassim population in Saudi Arabia.

Inclusion criteria: Female citizens of Qassim Province who are ≥ 18 years of age.

Exclusion Criteria: Female younger than 18 years of age. All age groups of males.

Data management and analysis plan

The current study aimed to evaluate the awareness level of women from Qassim Province about the role of epidural analgesia in labor pain management. Collected data were analyzed using SPSS Statistics v. 26 (IBM Corp., Armonk, NY), and results were presented as numbers and percentages. The study examined different associations between variables using various statistical analyses. Firstly, the frequencies and percentages of 15 questions concerning epidural analgesia were analyzed to make inferences about awareness. Secondly, the association between pregnancy history and awareness of epidural analgesia was analyzed using the chi-square test to compare the observed and expected statistics. A p-value of less than 0.05 was considered significant. 

Definition of awareness levels of epidural analgesia

This covers the questions asked by women using it for pain management during labor and the measures taken by health organizations to make women aware of the advantages of epidural analgesia, its availability, procedures, and possible complications.

## Results

This study received 520 responses, 37 of which were filtered out as being either responded to by males (most likely by the patient attendees) or by underage women (less than 18 years old). Subsequently, 483 responses were usable for analysis and inferential statistics, constituting 92.88% of the total respondents, who were all women and above the age of 18 years.

As shown in Table 1, 53.4% of the women had prior exposure to epidural analgesia, while 46.6% did not know about it (Q1). Among those who had prior exposure, 26.3% (n = 127) knew it from their friends or relatives, 13.5% (n = 65) got the information from media or readings, 5% (n = 24) from previous deliveries, 4.6% (n = 22) from talks at maternity clinics/hospitals, and 3.3% (n = 16) from pamphlets/brochures at maternity clinics/hospitals. Moreover, 52.6% (n = 254) never knew if epidural analgesia services were available at the local hospital, 34.4% (n = 166) confirmed the availability, and 13% (n = 63) denied the availability of this service (Q2). Most respondents (30%; n = 145) were unaware of the most effective means of pain control. Nevertheless, the other majority (29.2%; n = 141) named epidural analgesia the best available method (Q3). In Q4, the majority (38.3%; n = 185) were not indecisive about using epidural analgesia in the future, yet 34.8% (n = 168) showed their willingness to use it, and 26.9% (n = 130) denied using it in the future. Further, 40% (n = 193) of the women never requested epidural analgesia for delivery; only 14.9% (n = 72) requested epidural analgesia during labor (Q5). Regarding not requesting epidural analgesia for the labor, 12.2% (n = 59) said that they avoided it due to possible complications, while 6% (n = 29) thought it was not available (Q6). In Q7 about the request for epidural analgesia during labor, the majority (57.1%; n = 276) expressed that they should be informed about it before applying; among those willing to have it, the majority (21.5%; n = 104) wanted it to be introduced by the consultant doctor, and 21% (n = 101) wanted it to be introduced by nursing staff or anesthesiologists. In addition, 79.9% (n = 386) of the women revealed that they did not undergo the procedure after it was requested, while 20.1% (n = 97) underwent the procedure (Q8). Out of those who underwent epidural analgesia procedures, the majority (9.1%, n = 44) expressed that they did not feel any pain at all, while 5.8% (n = 28) reported most of the pain was relieved, and additionally, 2.7% (n = 13) reported having major pains gone, while only 0.8% (n = 4) expressed having no effect (Q9). Of those who underwent this procedure, 9.9% (n = 48) got it on time, 7.9% (n = 38) got it a little late, and 2.3% (n = 11) got it very late, actually near the delivery (Q10). In response to the Q10 follow-up, the majority (7.2%; n = 35) said that epidural analgesia was not available upon request, the medical team advised 4.6% (n = 22) that it was not suitable for them, and 2.7% (n = 13) said a doctor was not available for the procedure. Among those who underwent the epidural analgesia procedure, the majority (16.8%, n = 81) reported it to be a good experience, while only 3.3% (n = 16) reported it as a bad experience, and among those who reported bad experience, the majority (2.3%, n = 11) said it was too painful (Q11). The majority (35.4%; n = 171) of the women who had epidural analgesia reported that postpartum back pain was a possible complication, 34.8% (n = 168) reported injury to important organs, 13.7% (n = 66) reported breathing difficulties, and 9.7% (n = 47) reported immobility. Up to 1 day, 6.4% (n = 31) reported harmful effects on the fetus as a potential complication of having epidural analgesia for labor (Q12). Most women (30%; n = 145) were unsure if women generally use epidural analgesia during labor. However, the second majority (26.3%; n = 127) thought it is often used, 14.9% (n = 72) thought it is mostly used, 14.7% (n = 71) thought it is occasionally used, and 14.1% (n = 68) thought it is rarely used (Q13). Concerning the availability of epidural analgesia for labor, the majority (44.3%; n = 214) wanted it to be available for labor, 32.7% (n = 158) were indecisive, and 23% (n = 111) said it would not be available (Q14). Lastly, on the use of epidural analgesia in HA hospitals as compared with private hospitals, the majority (46.4%; n = 224) expressed that HA hospitals were not using it more than private hospitals, 32.1% (n = 155) were not aware of which had a higher percentage of use, and 21.5% (n = 104) considered that the HA use percentage was higher than the private hospitals (Q15).

**Table 1:** Summary of Replies to Questionnaire Multiple responses were allowed

Questions	N	%
Q1. Have you been exposed/introduced to epidural analgesia for pain control in labor?		
No	225	46.6
Yes	258	53.4
If yes		
From antenatal talks in the hospital/Maternal and Child Health Clinics	22	4.6
From experience in previous deliveries	24	5.0
From friends or relatives	127	26.3
From pamphlets/brochures in the antenatal clinic	16	3.3
From the media or readings	65	13.5
Q2. Do you know your hospital offers an obstetric epidural analgesia service?		
Don’t Know	254	52.6
Service Available	166	34.4
Service Not Available	63	13.0
Q3. What do you think is the most effective means of pain control in labor?		
Don’t Know	145	30.0
Epidural Analgesia	141	29.2
Muscular Pain Killing Injections	72	14.9
No Method is Effective	38	7.9
Others	9	1.9
Pain Killing Gas Inhalation	38	7.9
Patient Controlled IV Injections	40	8.3
Q4. In the future or if you are pregnant right now, will you request an epidural when in labor?		
No	130	26.9
Not Decided	185	38.3
Yes	168	34.8
Q5. Did you request an epidural when you were in labor?		
Had Caesarean / No Labor	47	9.7
No	193	40.0
No Previous Pregnancy	171	35.4
Yes	72	14.9
Q6. If you were not going to/did not request an epidural during your labor, which of the following reasons would have applied?		
I delivered too quickly	9	1.9
I did not need pain control	24	5.0
I did not think it was available/I am not eligible	29	6.0
I wanted the labor to progress naturally	44	9.1
I was worried about complications of epidural analgesia	59	12.2
I was worried about increased obstetric interventions	29	6.0
No Answer	276	57.1
Others	13	2.7
Q7. If you plan to request epidural analgesia when in labor, would you want the issue to be introduced formally during antenatal visits?		
No	207	42.9
Yes	276	57.1
If yes		
By means of a pamphlet that I can read	12	2.5
By means of a video that I can watch	35	7.2
During antenatal talks by the nursing staff	51	10.6
During the doctor’s consultation	104	21.5
In a special session by the anesthetist	50	10.4
No introduction is needed – I understand already	24	5.0
Q8. If you did request epidural analgesia in labor, did you actually undergo the procedure?		
No	386	79.9
Yes	97	20.1
Q9. How satisfied were you with the epidural procedure and its effects on your labor pains?		
Perfect. I did not have any pain at all	44	9.1
Satisfied. The epidural relieved major pains except for some minor pains	13	2.7
The epidural did not help at all	4	0.8
The epidural helped a bit only	8	1.7
Very satisfied. Helped relieve most of my pains	28	5.8
No Answer	386	79.9
Q10. Was the insertion of the epidural analgesia timely?		
It was inserted at exactly the time I wanted	48	9.9
It was inserted only after I had a lot of pain	38	7.9
It was inserted too late when I was about to deliver	11	2.3
No Answer	386	79.9
what was the reason you requested but did not receive an epidural?		
I got it	10	2.1
The doctor/nurse said it was not suitable for me	22	4.6
The epidural doctor was too busy to come	13	2.7
The quota for the service was full	16	3.3
The service was not available when I requested it	35	7.2
No Answer	387	80.1
Q11. How do you find the experience of your labor and delivery?		
Bad	16	3.3
Good	81	16.8
No Answer	386	79.9
If Bad		
I couldn 't feel the urge to push	3	0.6
I was anxious about the baby’s/my condition	2	0.4
It was too painful	11	2.3
Q12. Which of the following do you think are possible complications of epidural analgesia?*		
Breathing Difficulties	66	13.7
Harmful Effects on the Fetus	31	6.4
Immobility Up to 1 Day	47	9.7
Injury to Important Organs	168	34.8
Postpartum Back Pain	171	35.4
Q13. How commonly do you think epidural analgesia is used in women during their labor?		
For Most Women	72	14.9
Not sure	145	30.0
Occasionally, for specific indications	71	14.7
Often	127	26.3
Rarely	68	14.1
Q14. Do you think epidural analgesia should be available to all suitable obstetric patients going through labor?		
Don’t Know	158	32.7
No	111	23.0
Yes	214	44.3
Q15. Do you think that the percentage of obstetric patients who use epidural analgesia for pain control is higher in HA hospitals than in private hospitals?		
Don’t Know	155	32.1
No	224	46.4
Yes	104	21.5

**Figure 1:**
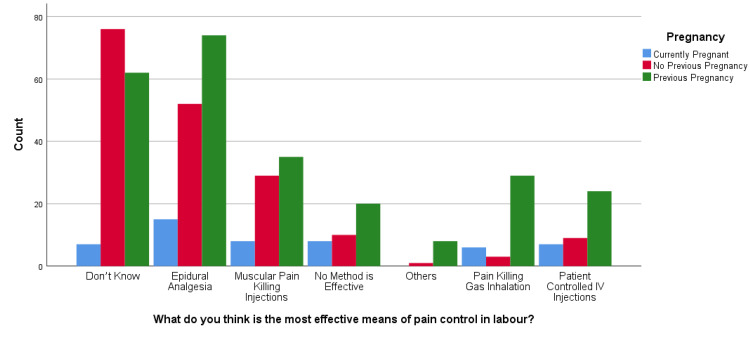
What do you think is the most effective means of pain control in labor?

**Figure 2:**
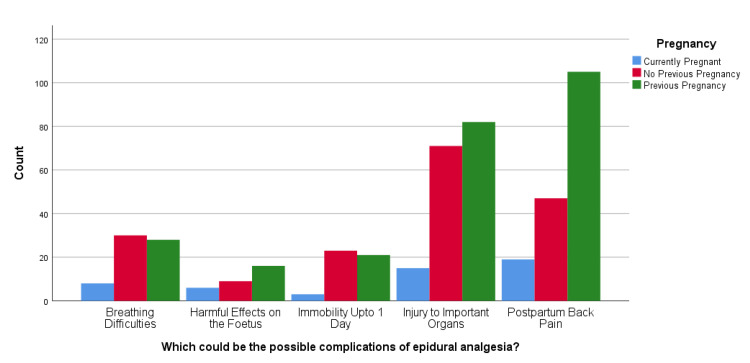
Which of the following do you think are possible complications of epidural analgesia?

**Figure 3:**
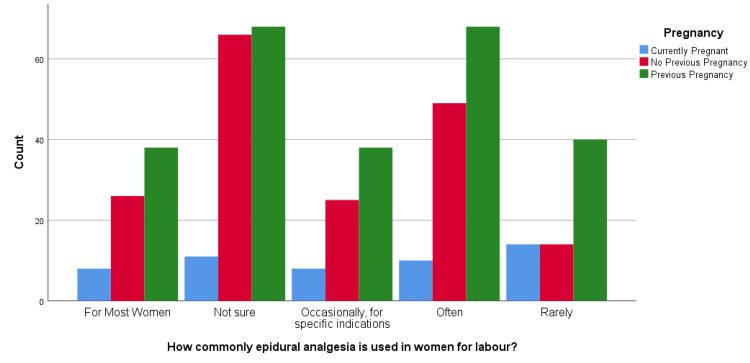
How commonly do you think epidural analgesia is used in women during their labor?

**Figure 4:**
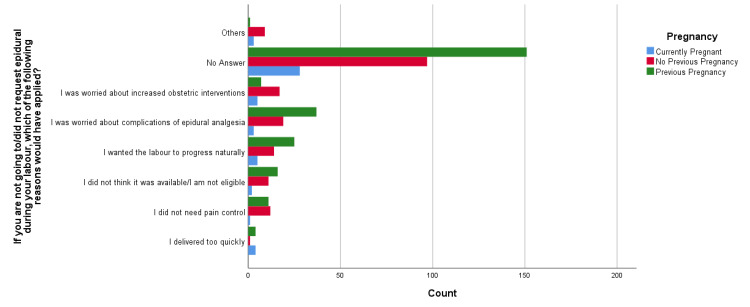
If you were not going to/did not request an epidural during your labor, which of the following reasons would have applied?

Aligned with the findings of the primary analysis, this study deemed it pertinent to explore further the association of pregnancy history with epidural analgesia awareness levels; see Table 2 for more details. For which chi-square tests were conducted, whose results revealed that there was a statistically significant association between "What do you think is the most effective means of pain control in labor?" and pregnancy history, X2(12) = 450.058, p < 0.001. Among the respondents, 29.2% (n = 141) found epidural analgesia to be the most effective method of pain control among all other alternatives. At the same time, post hoc analysis showed that most respondents (n = 74 out of 141) had used the same in the past. Among those who showed their unawareness, most of the women (n = 76 out of 145) had no previous pregnancy (see Figure 1). The analysis also revealed a statistically significant association between "Which of the following do you think are possible complications of epidural analgesia?" and pregnancy history (X2(8) = 17.523, p = 0.025). Postpartum back pain (35.4%; n = 171) was the major concern related to the use of epidural analgesia, and injury to important organs (34.8%, n = 168) was an almost equally concerning complication. Post hoc analysis showed that the majority of the women (n = 105 out of 171) reporting back pain had used it in a previous pregnancy; similarly, the majority (n = 82 out of 168) had a previous pregnancy and reported complications related to major organs (see Figure 2). Similarly, results showed a statistically significant association between "How commonly do you think epidural analgesia is used in women during their labor?" and pregnancy history, X2(8) = 17.928, p = 0.022. Of the respondents, 26.3% (n = 127) said often it is used, while 14.9% (n = 72) said it is used for most women, while post hoc analysis showed that the majority (n = 68 out of 127 and n = 38 out of 72, respectively) had been associated with previous pregnancy as compared with no pregnancy or first-time pregnancy (see Figure 3). Likewise, results revealed a significant association between "If you were not going to/did not request epidural during your labor, which of the following reasons would have applied?" and pregnancy history (X2(14) = 38.303, p < 0.001). The majority (12.2%; n = 59) were concerned about the complications of using epidural analgesia. Post hoc analysis revealed that, out of those fearing the complications, there was a major association with previous pregnancy (n = 37) compared to other pregnancy history types (see Figure 4).

**Table 2:** Association Between Pregnancy History and Epidural Analgesia Awareness Level

Question	Pregnancy			Total	X^2^(df)	p
	Currently Pregnant	No Previous Pregnancy	Previous Pregnancy			
Q3. What do you think is the most effective means of pain control in labor?					45.058 (12)	0.000
Don’t Know	7	76	62	145 (30%)		
Epidural Analgesia	15	52	74	141 (29.2%)		
Muscular Pain Killing Injections	8	29	35	72 (14.9%)		
No Method is Effective	8	10	20	38 (7.9%)		
Others	0	1	8	9 (1.9%)		
Pain Killing Gas Inhalation	6	3	29	38 (7.9%)		
Patient Controlled IV Injections	7	9	24	40 (8.3%)		
Q12. Which of the following do you think are possible complications of epidural analgesia?					17.523 (8)	0.025
Breathing Difficulties	8	30	28	66 (13.7%)		
Harmful Effects on the Fetus	6	9	16	31 (6.4%)		
Immobility Up to 1 Day	3	23	21	47 (9.7%)		
Injury to Important Organs	15	71	82	168 (34.8%)		
Postpartum Back Pain	19	47	105	171 (35.4%)		
Q13. How commonly do you think epidural analgesia is used in women during their labor?					17.928 (8)	0.022
For Most Women	8	26	38	72 (14.9%)		
Not sure	11	66	68	145 (30%)		
Occasionally, for specific indications	8	25	38	71 (14.7%)		
Often	10	49	68	127 (26.3%)		
Rarely	14	14	40	68 (14.1%)		
Q6. If you were not going to/did not request an epidural during your labor, which of the following reasons would have applied?					38.303 (14)	0.000
I delivered too quickly	4	1	4	9 (1.9%)		
I did not need pain control	1	12	11	24 (5%)		
I did not think it was available/I am not eligible	2	11	16	29 (6%)		
I wanted the labor to progress naturally	5	14	25	44 (9.1%)		
I was worried about complications of epidural analgesia	3	19	37	59 (12.2%)		
I was worried about increased obstetric interventions	5	17	7	29 (6%)		
No Answer	28	97	151	276 (57.1%)		
Others	3	9	1	13 (2.7%)		

## Discussion

There are mixed outputs regarding the awareness levels of epidural analgesia among Saudi women in Qassim. Although the majority (53.4%) were aware of the role of epidural analgesia in labor pain, their source of information was mainly their close circle of relationships. Only 7.9% of the awareness was available to them from clinics or hospitals, compared to a total of 31.3% from personal sources; this shows a significant gap regarding the efforts of medical organizations to raise awareness of epidural analgesia. Moreover, 65.6% of respondents reported that either there was no service available or they were unaware of it, which is quite concerning and poses a serious question about the service provider's credibility. Another gap was found when the majority of the women (30%) showed their unawareness of the most effective method of pain control. However, it was a good discovery that 29.2% of women found epidural analgesia to be the best available method to cope with labor pain; this shows the effectiveness of this procedure. If indecisive women are not considered, then it is safe to say that most women (38.3%) will also be using epidural analgesia in the future. A significant percentage of women (34.8%) opted out. Statistics on the request for the procedure showed poor demand for epidural analgesia. Only 14.9% of women requested epidural analgesia during labor, and 12.2% of women said they did not request it due to possible complications.

Further, 57.1% indicated that it should be introduced for application by the medical consultant, which shows that women would like professionals to be their guides instead of their personal sources. Regarding the application of epidural analgesia, only 20.1% underwent the procedure, leaving room for more investigations to determine whether it was due to personal or operational reasons.

Those who underwent epidural analgesia procedures (17.6% jointly) expressed that the procedure was effective enough in completely or partially relieving their labor pain. The efficiency of the service delivery was observable, as most of the women (9.9%) received it on time. Yet, there remains room for improvement, considering 10.2% jointly reported major or minor delays in the service.

On the availability of the epidural analgesia service, 11.8% of respondents jointly reported that this service or the concerned doctor was not available, which is also an improbable area. The majority (16.8%) reported receiving epidural analgesia as an excellent experience; however, a minority (3.3%) reported it as a bad experience due to it being too painful. Regarding the complications, two significant findings concern postpartum back pain and injury to important organs. These findings can be correlated with those of To, W. W. (2007) [10] and warrant more in-depth exploration, which could help build a national-level pain management inventory. Another lack was discovered as most of the women (30%) were unsure about other women who had been using epidural analgesia in general and asked for more intensive campaigns to raise awareness. Despite the varying responses regarding the advantages and disadvantages of epidural analgesia, most women (44.3%) wanted it to be available for their next labor. Lack of awareness was further highlighted by the fact that the majority (46.4%) were unaware of which type of hospitals-HA or private-offered epidural analgesia.

Additionally, 32.1% (n = 155) were unaware of the extent to which this service was available at these hospitals, which created doubts about the health organizations' credibility and effort levels. Finally, this study found significant associations between awareness levels of epidural analgesia and pregnancy history. Women who had given birth before reported epidural analgesia as the most effective means of pain control (p < 0.001), postpartum back pain as the major complication (p = 0.025), being used by most other women (p = 0.022), and the potential for epidural analgesia to yield complications (p < 0.001). This study also has some limitations. First, this study lacks prior research studies on the topic, as this is the first study conducted in Al Qassim Province about this subject. Second, the data were self-reported, so we must take the people's word, as some people may not even remember the whole experience.

In 2017, a cross-sectional hospital-based study was conducted at King Faisal Specialist Hospital and Research Center on the awareness of epidural analgesia among pregnant women receiving antenatal care at the hospital's clinics. Questionnaires were distributed among the pregnant women, and 384 responses were collected. The majority of the respondents were aged 20-35 years. In general, this study's results are similar to ours regarding the current level of awareness of epidural analgesia among women and the need to improve it. The results demonstrated a clear lack of knowledge about epidural analgesia in Saudi Arabia and the pressing need to increase it. Generally, women in other countries are more aware of this procedure. Furthermore, the study highlighted that women between 21 and 35 years were more likely to undergo epidural analgesia during labor than women over 35, indicating increased awareness among the younger age group. The results also demonstrated that women previously undergoing epidural analgesia were more likely to opt for it again for future labor [12].

In supporting our study result, a study by Minhas et al. in 2003 in Pakistan with 448 patients found that the most stated fear about EA was back pain (24%), which matches our result as the most common possible complication (34%). Regarding the source of knowledge about EA, our study's primary source of information was the close circuit of relationships, which was the same as in the Pakistan study and was friends or relatives [13].

In our study, only 7.9% of the awareness about epidural anesthesia was available to them from clinics or hospitals. In 2017, Manisha Thakur, Nidhi Sagar, and Pooja Tandon conducted a study in India. They found that 46.7% of expectant mothers had below-average levels of knowledge, 35% had a moderate level of knowledge, and 18.3% had a good level of knowledge. In both studies, there is a significant gap regarding the efforts of medical organizations to raise awareness of epidural analgesia [14].

In 2022, a group of researchers conducted a study in Jazan, Saudi Arabia, and found that the knowledge and awareness of epidural analgesia among pregnant women were deficient compared to our study's results [15]. This difference might be because of educational level differences in comparison between the two regions and the health education program in the healthcare centers. The cultural differences may also affect the study's validity. Future research can explore more such associations to widen the scope of this field of study.

## Conclusions

The present study revealed mixed awareness levels about epidural analgesia among Saudi women from the Al Qassim area. While most women were aware of the role of epidural analgesia in labor pain, their sources of information were mainly personal networks. Information received from clinics or hospitals was found to be limited. Furthermore, the study found a lack of availability for the service, which raises questions about the service provider's credibility. The procedure's effectiveness is evident from the fact that a significant number of women found it to be the best available method to cope with labor pain. However, the demand for the procedure was found to be low due to concerns about possible complications.

This study highlights the importance of timely and efficient delivery of the service, and it is important because it addresses the shortcomings in this area. In terms of future actions, it is recommended that medical organizations take steps to improve the availability and accessibility of epidural analgesia by providing information to pregnant women during prenatal visits. In addition, more intensive awareness campaigns are needed to increase the demand for the procedure and address the concerns of women regarding possible complications arising from the procedure. Further research is required to explore the associations between awareness levels of epidural analgesia and pregnancy history and the complications associated with the procedure. Overall, the study highlights the need for a concerted effort by healthcare providers to improve the awareness and uptake of epidural analgesia in the Al Qassim Province and beyond.
